# Immediate Implant-Based Breast Reconstruction: Analysis of 787 Cases and Definition of Our Decision-Making Algorithm

**DOI:** 10.1007/s00266-026-05900-5

**Published:** 2026-05-12

**Authors:** Federico Taraschi, Liliana Barone Adesi, Nicole Ratti, Giuseppe Visconti, Luca Ricci, Lorenzo Scardina, Gianluca Franceschini, Marzia Salgarello

**Affiliations:** 1 UO Chirurgia Plastica, Dipartimento per la Salute della Donna, del Bambino e di Sanità Pubblica, Fondazione Policlinico Universitario “Agostino Gemelli” IRCCS - Università Cattolica del “Sacro Cuore”, Largo Agostino Gemelli 8, 00168 Rome, Italy; 2 Breast Unit, Departement of Women, Children and Public Health Sciences, Fondazione Policlinico Universitario Agostino Gemelli IRCCS, 00168 Rome, Italy

**Keywords:** Breast reconstruction, Implant-based breast reconstruction, Prepectoral reconstruction, Submuscular direct-to-implant, Tissue expander reconstruction

## Abstract

**Background:**

Immediate implant-based breast reconstruction (IBBR) following nipple-sparing (NSM) or skin-sparing mastectomy (SSM) increasingly requires individualized technique selection. Choosing among prepectoral direct-to-implant (DTI), submuscular DTI, and two-stage expander reconstruction remains complex. This study analyses outcomes from a large single-centre cohort to define a pragmatic, evidence-based decision-making algorithm.

**Methods:**

A retrospective monocentric review included all consecutive IBBR procedures performed between 2018 and 2024. Patients were allocated to three groups: prepectoral DTI (n = 601), submuscular DTI (n = 142), and two-stage expander reconstruction (n = 44). Demographic, oncologic, and surgical variables were collected. Major complications were evaluated using unadjusted analyses and inverse-probability-of-treatment weighting. A dedicated subgroup analysis was performed for patients receiving postmastectomy radiotherapy (PMRT).

**Results:**

A total of 1,093 breasts were reconstructed. Weighted analysis showed similar overall major-complication rates for prepectoral and submuscular DTI (7.3% vs 8.4%, *p* = 0.278), while expanders demonstrated the lowest adjusted risk (0.7%). Within the PMRT subgroup, prepectoral DTI did not show increased infection, dehiscence, or seroma compared with the other techniques; infection was significantly lower than in submuscular DTI (2.7% vs 14.3%, *p* = 0.020). Capsular contracture was markedly less frequent in prepectoral DTI and remained significantly reduced after multivariable adjustment (OR 1 [reference]; OR 9.03 for submuscular DTI; OR 3.25 for expanders).

**Conclusions:**

Our resulting algorithm offers a structured, patient-specific approach to optimise outcomes in immediate implant-based breast reconstruction.

**Level of Evidence IV:**

This journal requires that authors assign a level of evidence to each article. For a full description of these Evidence-Based Medicine ratings, please refer to the Table of Contents or the online Instructions to Authors www.springer.com/00266.

## Introduction

Breast cancer is the most common malignant neoplasm among women worldwide, with over 2.2 million new cases diagnosed in 2022. [1]. Breast-conserving surgery is feasible in 70–80% of patients as part of the primary treatment strategy [2, 3]. However, in approximately 30% of cases, mastectomy remains essential to ensure oncological safety. The change in body image following mastectomy has a significant impact on the patients’ psycho-physical well-being [4]. In this context, breast reconstruction has become fundamental in reducing morbidity and improving patients' quality of life. Breast reconstruction can be immediate or delayed, the latter being planned after adjuvant treatments [5, 6]. Both approaches can be performed using autologous tissues or prosthetic implants [7]. Autologous reconstruction involves the use of pedicled flaps, free flaps, fat grafting, reverse expansion techniques, or hybrid approaches combining prosthetic and autologous methods [8–12]. Immediate prosthetic reconstruction can be performed in a one-stage or two-stage manner, using a tissue expander followed by a permanent implant [13]. The implant may be positioned in either a subpectoral, prepectoral, or dual plane [14, 15]. According to the 2023 report by the American Society of Plastic Surgeons, immediate prosthetic reconstruction remains the most common approach, with a growing preference for the prepectoral technique over the submuscular one [16]. Prepectoral breast reconstruction (PPBR) has rapidly gained popularity due to its favourable aesthetic outcomes, minimal functional impairment, and low complication rates [17]. Unlike the submuscular technique, often associated with animation deformity, capsular contracture, and suboptimal cosmetic outcomes, PPBR circumvents many of these issues [18, 19]. In PPBR, implants are typically placed in the subcutaneous plane and often wrapped in an acellular dermal matrix (ADM) or synthetic mesh [20–22], alternatively, polyurethane-coated implants have been explored for their potential to reduce certain complications and eliminate the need for ADM/meshes, although comparative evidence remains limited [23–26]. To achieve optimal breast reconstruction, it is essential to clearly define reconstructive goals, including restoration of the breast footprint, envelope, and conus, key elements in recreating a natural and harmonious breast shape [27].

Finally, selecting the ideal reconstructive technique requires a careful evaluation of multiple factors, including the type of mastectomy, adjuvant therapies, patient-specific anatomy and clinical conditions, personal preferences, and logistical considerations such as surgical expertise and available resources. These elements significantly influence short-, medium-, and long-term outcomes.

Predictable patient and breast specific risk factors increase complications after immediate implant-based reconstruction, including smoking, obesity, and large breast or implant size [28]. The impact of neoadjuvant chemotherapy (NACT) and prior irradiation remains debated: NACT can impair immune response and wound healing [29–31]; yet several series report comparable expander/implant loss and reconstruction completion after NACT [32–34]. A meaningful subset of women treated with breast-conserving surgery and radiotherapy later require mastectomy due to recurrence, a new primary tumor on the same breast or genetic risk; ipsilateral breast tumor recurrence (IBTR) accounts for 5–15% of all cancer recurrences in women treated with breast conservative treatment [35, 36]. Irradiation induces dermal fibrosis and subcutaneous vascular changes, heightening adverse reconstructive outcomes [37]. Complications remain more frequent even with autologous reconstruction; some patients still prefer implants, whereas others are not candidates for autologous procedures [38–40]. Evidence on the specific impact of NACT or prior radiotherapy on morbidity after direct-to-implant (DTI) reconstruction is limited by small cohorts, and the optimal reconstructive choice remains debated [41–43]

Postmastectomy radiotherapy (PMRT) improves survival and locoregional control but compromises reconstruction by damaging dermal microvasculature and inducing soft-tissue fibrosis, increasing infection, capsular contracture, and implant loss, and worsening aesthetics versus no PMRT [44–46]. These changes are difficult to correct and may result in persistently suboptimal cosmetic outcomes [47, 48]. Nonetheless, PMRT remains integral to oncologic care and must inform reconstructive planning [49].

Mastectomy skin-flap viability is critical. Intraoperative assessment may be clinical or augmented with indocyanine green angiography (ICGA) for real-time perfusion mapping [50, 51], which is associated with reduced flap necrosis and enables targeted debridement and adjustment of technique and implant size to minimize complications [50].

As DTI gains adoption, an evidence-based appraisal of comparative risks is needed to support shared decision-making. The aim of this study is to analyze the outcomes of 787 patients according to the surgical technique and to define our decision-making algorithm.

## Materials and Methods

This retrospective, monocentric study included all patients who underwent nipple-sparing mastectomy (NSM) or skin-sparing mastectomy (SSM) with immediate implant-based breast reconstruction between 2018 and 2024 at our institution. Exclusion criteria were delayed reconstruction, autologous or hybrid procedures, skin-reducing mastectomy, and incomplete clinical documentation, including missing intraoperative data or insufficient postoperative follow-up.

Patients were stratified into three groups according to the reconstructive approach: prepectoral direct-to-implant (DTI) reconstruction, submuscular DTI reconstruction, and two-stage submuscular reconstruction involving a tissue expander followed by definitive implant placement. Once the tissue expander was replaced with the implant, the patients were excluded from follow-up. Group assignment was based on oncologic requirements, patient-specific anatomical and clinical characteristics, shared decision-making with the patient, and surgeon preference.

Data collection included demographic variables (age, body mass index, smoking status), oncologic treatments (neoadjuvant and adjuvant chemotherapy and radiotherapy), and surgical characteristics (type and laterality of mastectomy; mastectomy incision, selected according to each patient’s oncologic, clinical, and anatomical features; mastectomy flap thickness; implant plane; use of acellular dermal matrix [ADM]; type and volume of implant; and mastectomy specimen weight), as well as postoperative complications. All patients underwent preoperative mammography.

Complications were categorized as early (within 30 days of surgery) or late (after 30 days), and included flap necrosis, wound dehiscence, infection, implant extrusion, red breast syndrome, capsular contracture, rippling, hematoma, and seroma.

Collected data were inserted in a database on Microsoft Excel (Microsoft Corp, Redmond, WA, USA) and then analyzed using R statistical software (The R Foundation for Statistical Computing) via the RStudio interface (Version 1.4.1103 © 2009-2021 RStudio, PBC). Quantitative variables were expressed as means and standard deviations or medians and interquartile ranges, according to data distribution. Categorical variables were presented as absolute frequencies and percentages. Inter-group comparisons were performed using the chi-square test or Fisher’s exact test for categorical variables, and the t-test for continuous variables, depending on normality. Multivariate regression analysis was performed to identify independent predictors of reconstructive outcomes. A p-value < 0.05 was considered statistically significant.

## Results

**Fig. 1:**
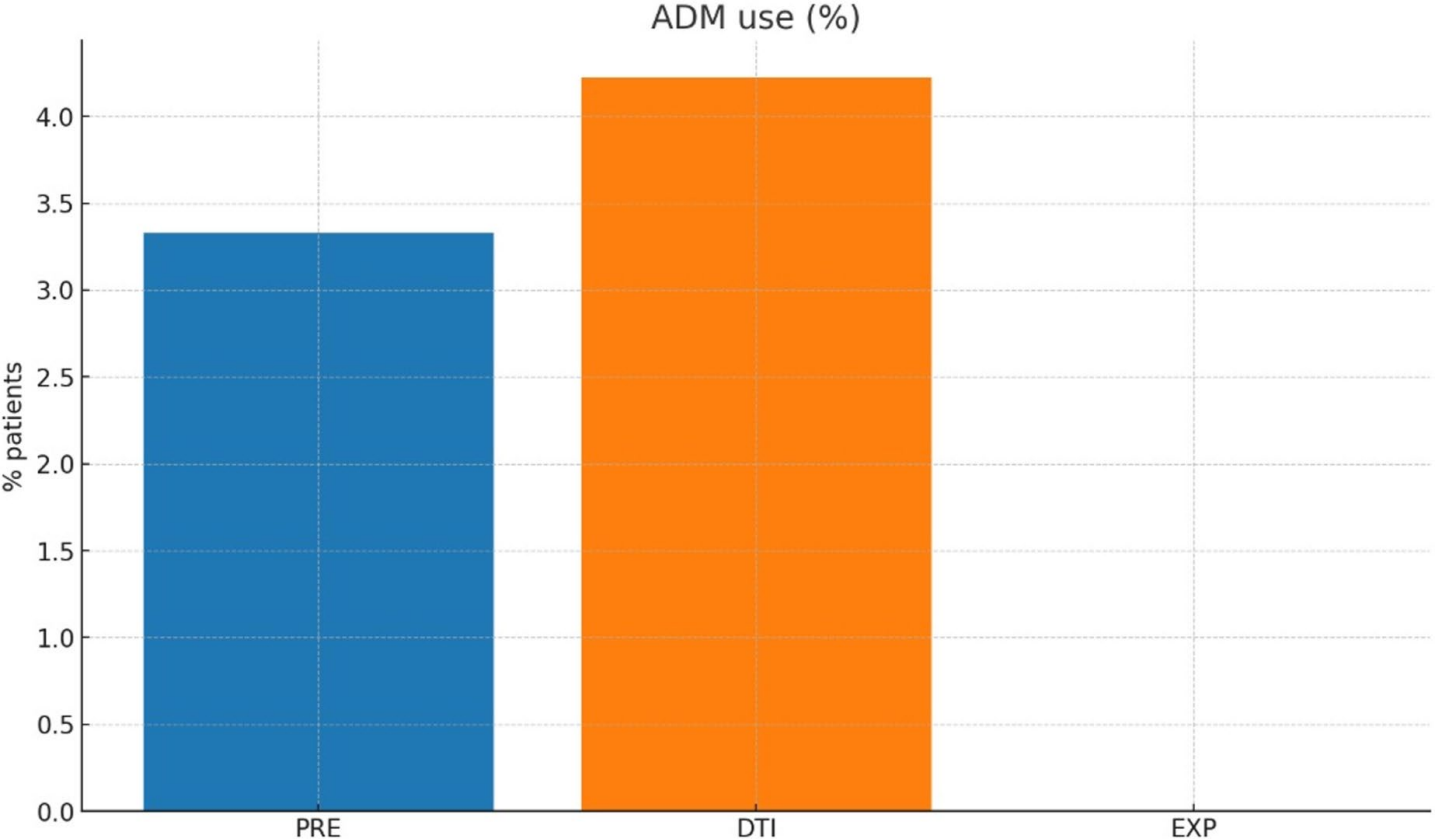
Percentage (%) of ADM (acellular dermal matrix) use in the three groups analyzed: prepectoral direct-to-implant reconstruction (PRE), submuscular direct-to-implant reconstruction (DTI), and two-stage submuscular reconstruction with a tissue expander (EXP)

**Fig. 2:**
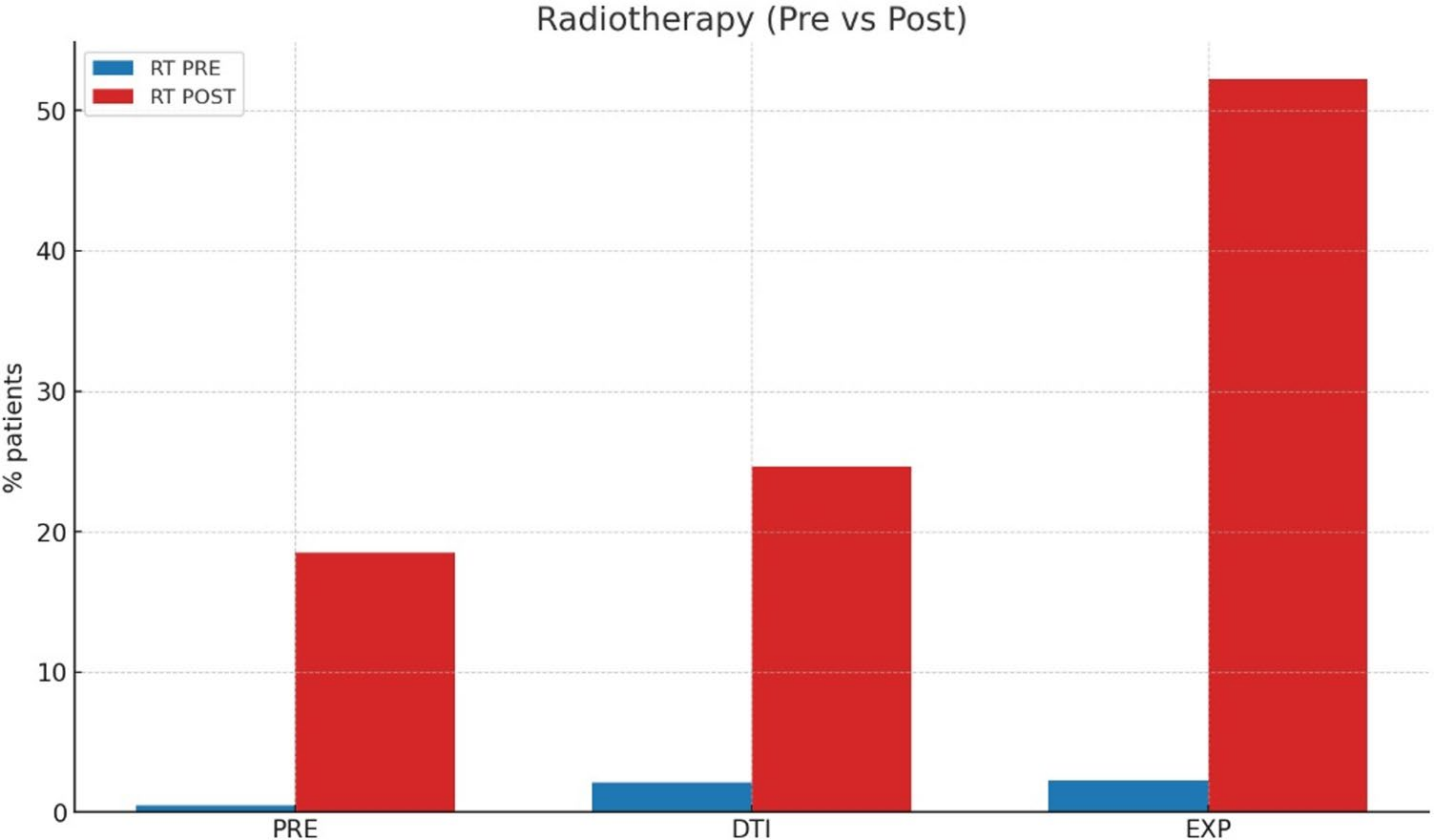
Distribution of radiotherapy (RT) administered before (RT PRE, blue) and after (RT POST, red) reconstruction across the three groups: prepectoral direct-to-implant reconstruction (PRE), submuscular direct-to-implant reconstruction (DTI), and two-stage submuscular reconstruction with a tissue expander (EXP).

**Fig. 3:**
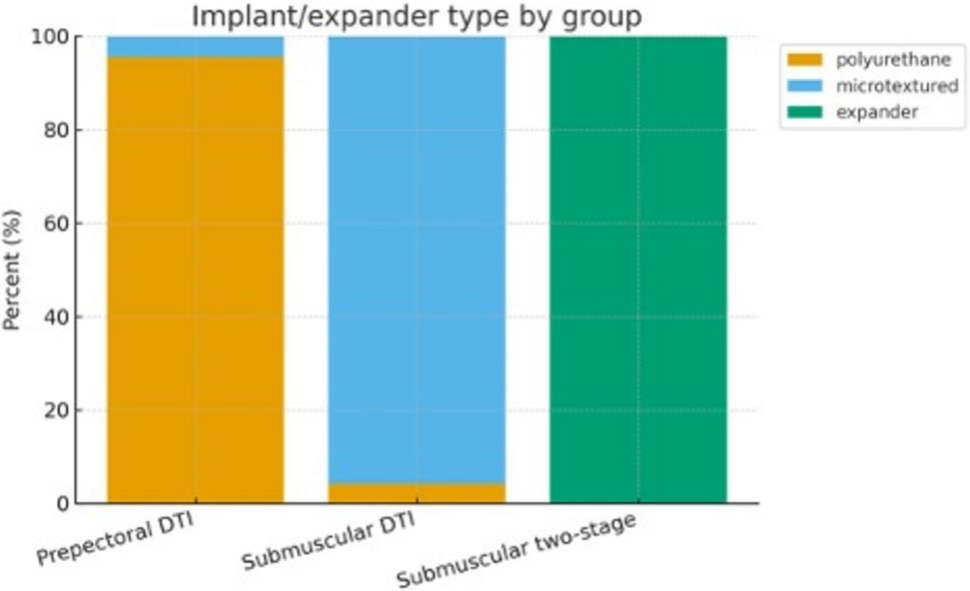
Distribution of implant/expander type across the three reconstruction groups: prepectoral direct-to-implant (DTI) reconstruction, submuscular direct-to-implant (DTI) reconstruction, and submuscular two-stage reconstruction with a tissue expander. Bars represent the proportion of polyurethane implants, microtextured implants, and expanders used in each group.

**Fig. 4:**
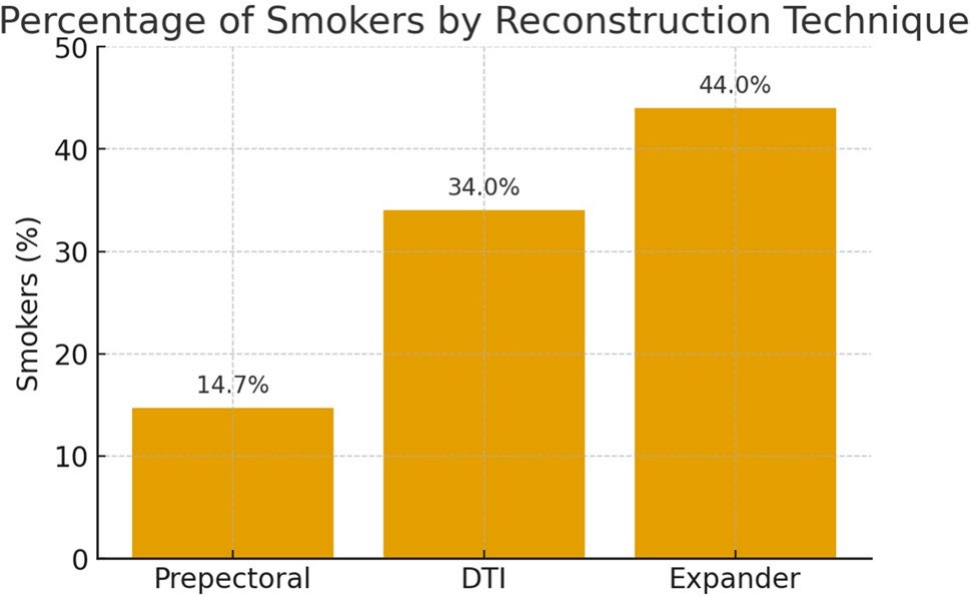
Percentage (%) of smokers across the three groups analyzed: prepectoral direct-to-implant reconstruction (Prepectoral), submuscular direct-to-implant reconstruction (DTI), and two-stage submuscular reconstruction with a tissue expander (Expander).

**Fig. 5:**
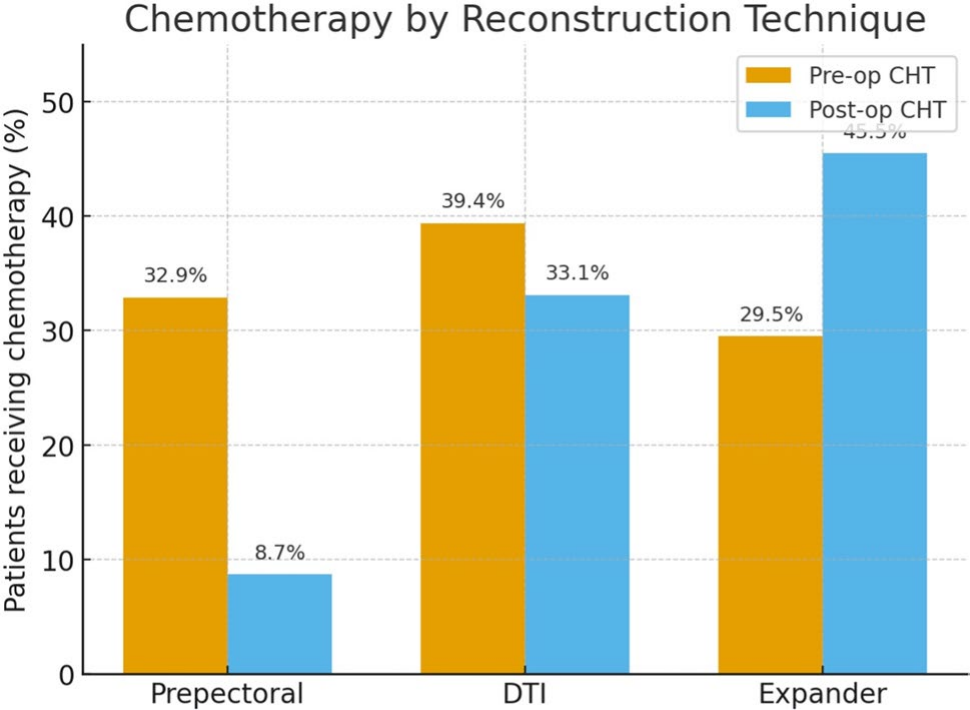
Distribution of chemotherapy (CHT) administered before (pre-op CHT, orange) and after (post-op CHT, blue) reconstruction across the three groups: prepectoral direct-to-implant reconstruction (prepectoral), submuscular direct-to-implant reconstruction (DTI), and two-stage submuscular reconstruction with a tissue expander (Expander).

**Fig. 6:**
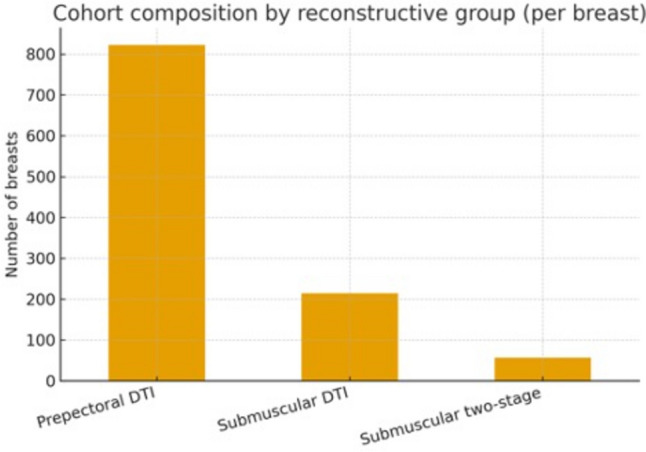
Cohort composition by reconstructive group (per breast). The study included 822 prepectoral direct-to-implant (DTI) reconstructions, 214 submuscular DTI reconstructions and 57 submuscular two-stage expander-based reconstructions.

A total of 787 patients who underwent immediate implant-based breast reconstruction after nipple- or skin-sparing mastectomy (705 NSM and 82 SSM) (2018–2024) were included (Figs. 1, 2, 3, 4, 5). Prepectoral reconstruction was performed in 601 patients, while 186 underwent submuscular reconstruction, either DTI (n = 142) or two-stage expander (n = 44). On a per-breast basis, a total of 1,093 breasts were reconstructed: prepectoral DTI (n = 822), submuscular DTI (n = 214), and submuscular two-stage (n = 57) (Fig. 6). Baseline features were broadly comparable (Table 1): mean age was 49.0 years in prepectoral versus 44.1 in DTI and 45.6 in expander; BMI 23.6 versus 20.8 versus 22.2 kg/m^2^. Smoking was most frequent in expander patients (44.0%) followed by submuscular DTI (34.0%) and prepectoral DTI (14.7)%. (Fig. 4) Preoperative chemotherapy were given in 32.9% of prepectoral, 39.4% of submuscular DTI, and 29.5% of expander patients and postoperative radiotherapy were given in 8.7% of prepectoral, 33.1% of submuscular DTI, and 45.5% of expander patients (Fig. 5); postoperative radiotherapy (PMRT) occurred in 18.5% of prepectoral DTI, 25.2% of submuscular DTI, and 52.3% of expanders (Fig. 2). From a technical standpoint, 95.8% of prepectoral cases received polyurethane-coated implants, whereas submuscular reconstructions were predominantly micro-textured (96.7%) (Fig. 3) ADM use was uncommon (prepectoral DTI 3.3%, submuscular DTI 4.2%, expander 0%) (Fig. 1). Mastectomy flap thickness was highest in the prepectoral group (mean 1.02 cm; expander 0.40 cm; not consistently recorded for DTI) (Table 2).

**Table 1:** Baseline characteristics of patients according to reconstruction technique

Characteristic	Prepectoral (n = 601)	DTI (n = 142)	Expander (n = 44)
Age (years)	49.0 ± 9.9	44.1 ± 7.6	45.5 ± 9.9
BMI (kg/m^2^)	23.6 ± 4.0	20.8 ± 2.3	22.2 ± 2.7
Smokers	14.7%	34.0%	43.2%
Pre-op chemotherapy	32.9%	39.4%	29.5%
Post-op chemotherapy	8.7%	33.1%	45.5%
Post-op radiotherapy	18.5%	25.2%	52.3%
ADM use	3.3%	6.3%	0.0%

Values are presented as mean ± standard deviation or percentage (%).

**Table 2:** Patient demographic and clinical characteristics according to the type of breast reconstruction

Characteristic	Prepectoral (number of patients = 601; number of breasts = 822)		DTI (number of patients = 142, number of breasts = 214)		Expander (number of patients = 44; number of breasts = 57)	
	Percentage (%)	Absolute value	Percentage (%)	Absolute value	Percentage (%)	Absolute value
Age (years)	49.0 ± 9.9		44.1 ± 7.6		45.5 ± 9.9	
BMI (kg/m^2^)	23.6 ± 4.0		20.8 ± 2.3		22.2 ± 2.7	
Smokers	14.7%	88.0	34.5%	49	43.2%	19
Pre-op chemotherapy	32.9%	198.0	39.4%	56.0	29.5%	13.0
Post-op chemotherapy	8.7%	52.0	33.1%	47.0	45.5%	20.0
Pre-op radiotherapy	0.5%	3.0	2.1%	3.0	2.3%	1.0
Post-op radiotherapy	18.5%	111.0	25.2%	35.0	52.3%	23.0
ADM use	3.3%	20.0	6.3%	9.0	0.0%	0.0
NSM right	27.3%	164.0	21.1%	30.0	25.0%	11.0
NSM left	30.1%	181.0	23.2%	33.0	27.3%	12.0
Bilateral NSM	31.6%	190.0	50.0%	71.0	29.5%	13.0
SSM right	3.8%	23.0	3.5%	5.0	4.5%	2.0
SSM left	5.8%	35.0	1.4%	2.0	13.6%	6.0
Bilateral SSM	1.3%	8.0	0.7%	1.0	0.0%	0.0
Average implant volume right (cc)		285.78		320.81		240.0
Average implant volume left (cc)		301.53		307.23		350.0
Average mastectomy wight right (g)		241.86	–	–	–	–
Average mastectomy wight left (g)		262.36	–	–	–	–
Polyurethane implant	95.8%	766.0	3.2%	7.0	0.0%	0.0
Microtextured implant	4.2%	34.0	96.7%	207.0	–	–
Average mastectomy flap thickness right (cm)		1.0	–	–		0.4
Average mastectomy flap thickness left (cm)		1.03	–	–		0.4
Mastectomy flap necrosis	1.3%	8.0	2.1%	3.0	0.0%	0.0
Dehiscence	1.7%	10.0	2.1%	3.0	0.0%	0.0
Infection	1.8%	11.0	4.2%	6.0	2.3%	1.0
Implant extrusion	0.7%	4.0	0.7%	1.0	0.0%	0.0
Red breast syndrome	0.5%	3.0	0.0%	0.0	0.0%	0.0
Capsular contraction	20.0%	120.0	21.8%	31.0	18.2%	8.0
Rippling	1.8%	11.0	2.8%	4.0	2.3%	1.0
Hematoma	0.7%	4.0	0.0%	0.0	2.3%	1.0
Seroma	2.8%	17.0	2.8%	4.0	0.0%	0.0

Data are presented as percentages (%) and absolute values, comparing prepectoral direct-to-implant reconstruction (n = 601 patients, 822 breasts), submuscular direct-to-implant reconstruction (DTI; n = 142 patients, 214 breasts), and two-stage submuscular reconstruction with a tissue expander (n = 44 patients, 57 breasts). Variables include patient demographics, oncologic treatments, surgical characteristics, implant types and volume and postoperative complications.

In adjusted multinomial models (baseline = prepectoral), PMRT independently increased the odds of selecting an expander (RRR≈4.10, *p* = 0.0001), while its association with submuscular DTI versus prepectoral was weaker and not significant (*p* = 0.081). Lower BMI (RRR ≈ 0.72 per kg/m^2^, *p* < 0.001) and younger age (RRR≈0.96 per year, *p* = 0.016) favored submuscular DTI. Nevertheless, a calendar-year model showed a clear shift toward prepectoral reconstructions over time (OR ≈ 1.3 per year, p≈0.02). (Table 3)

**Table 3:** Adjusted multinomial models (baseline =  prepectoral) for technique selection.

Comparison	Variable	RRR (95% CI)	*p*
DTI versus PRE	age	0.96 (0.94–0.98)	0.001
DTI versus PRE	bmi	0.72 (0.66–0.79)	0.0001
DTI versus PRE	smoke	0.94 (0.52–1.70)	0.831
DTI versus PRE	cht_pre	1.18 (0.76–1.84)	0.458
DTI versus PRE	cht_post	5.30 (3.17–8.85)	0.000
DTI versus PRE	rt_post	1.14 (0.68–1.90)	0.621
EXP versus PRE	age	0.98 (0.94–1.01)	0.192
EXP versus PRE	bmi	0.87 (0.78–0.97)	0.010
EXP versus PRE	smoke	2.45 (1.12–5.35)	0.025
EXP versus PRE	cht_pre	0.67 (0.32–1.41)	0.295
EXP versus PRE	cht_post	7.16 (3.52–14.57)	0.0001
EXP versus PRE	rt_post	4.10 (2.07–8.14)	0.0001

Relative risk ratios (RRR) with 95% confidence intervals (CI) and *p*-values are reported for comparisons between submuscular direct-to-implant reconstruction (DTI) and two-stage submuscular reconstruction with a tissue expander (EXP) versus prepectoral direct-to-implant reconstruction (PRE) as the reference category.

Variables included in the model were age, body mass index (BMI), smoking status, preoperative chemotherapy (cht_pre), postoperative chemotherapy (cht_post), and postoperative radiotherapy (rt_post).

Using a patient-level composite (infection, dehiscence, seroma, hematoma, implant extrusion), unadjusted rates were 7.5% for prepectoral, 8.5% for submuscular DTI, and 4.5% for expanders. After inverse-probability weighting (age, BMI, smoking, pre/post chemotherapy, PMRT, ADM), submuscular DTI had a slightly higher weighted risk than prepectoral (8.4% vs 7.3%, risk difference + 1.0 percentage point, *p* = 0.278), whereas expander had a lower weighted risk (0.7% vs 7.9%, risk-difference −7.4 pts, p<0.001); expander estimates are based on fewer complete cases (Table 4). In the PMRT subgroup (patients who actually received radiotherapy), the composite rate was 13.5% for prepectoral (15/111), 17.1% for submuscular DTI (6/35), and 8.7% for expanders (2/23); prepectoral versus expander (*p* = 0.73) and prepectoral versus submuscular DTI (*p* = 0.45) were not statistically different.

**Table 4:** Primary outcome—major complications: unadjusted and IPTW-adjusted analysis.

Measure	Prepectoral	DTI	Expander
Unadjusted rate (%)	7.5	8.5	4.5
IPTW weighted risk (%)	7.3	8.4	0.7
Risk difference DTI−PRE (pp)		1.0	
Risk difference EXP−PRE (pp)			− 7.2
p (RD) DTI−PRE		0.278	
p (RD) EXP−PRE			3.42e-12

Unadjusted complication rates and inverse probability of treatment weighting (IPTW)–adjusted risks are reported for prepectoral direct-to-implant reconstruction (Prepectoral), submuscular direct-to-implant reconstruction (DTI), and two-stage submuscular reconstruction with a tissue expander (Expander). Risk differences (RD) between techniques are expressed in percentage points (pp), with corresponding *p*-values for comparisons of DTI versus prepectoral (PRE) and expander (EXP) versus prepectoral reconstructions.

In the post-mastectomy radiotherapy (PMRT) subgroup, surgical-site events were uncommon with prepectoral reconstruction and generally less frequent than with DTI techniques. Infections occurred in 2.7% of prepectoral cases versus 4.3% with tissue expanders (not significant, *p* = 0.534) and 14.3% with submuscular DTI (significantly higher than prepectoral, *p* = 0.020) (Table 5). Wound dehiscence was observed in 0.9% of prepectoral reconstructions, 0% with expanders (*p* = 1.000), and 5.7% with DTI, reflecting a borderline increase for submuscular DTI relative to prepectoral DTI (*p* = 0.143). Seroma was documented in 7.2% of prepectoral procedures and in 0% of both expander and submuscular DTI cohorts; these differences were not statistically significant (all *p* ≥ 0.13). (Table 5).

**Table 5:** Postmastectomy radiotherapy (PMRT) subgroup—pairwise comparisons between reconstruction techniques.

Outcome	Prepectoral e/n	DTI e/n	Expander e/n	*p* (PRE vs DTI)	*p* (PRE vs EXP)
Infection	3/111	5/35	1/23	0.020	0.534
Dehiscence	1/111	2/35	0/23	0.143	1.000
Seroma	8/111	0/35	0/23	0.199	0.350

Incidence of postoperative complications (infection, dehiscence, and seroma) is reported as events per total cases (e/n) for prepectoral direct-to-implant reconstruction (Prepectoral), submuscular direct-to-implant reconstruction (DTI), and two-stage submuscular reconstruction with a tissue expander (Expander). *p*-values correspond to pairwise statistical tests comparing prepectoral (PRE) versus DTI and prepectoral versus expander (EXP) groups.

Capsular contracture showed marked divergence by technique. Crude rates in PMRT were 22.5% for prepectoral reconstruction (25/111), 62.9% for submuscular DTI (22/35), and 39.1% for expanders (9/23), with a highly significant overall difference (global χ^2^ *p* =  4.61 × 10⁻^5^). Pairwise comparisons indicated a substantially higher contracture rate with submuscular DTI versus prepectoral (*p* = 4.3×10⁻^5^), whereas the expander–prepectoral difference did not reach significance (*p* = 0.159). After multiple imputation and multivariable logistic regression adjusting for age, BMI, smoking status, neoadjuvant/adjuvant chemotherapy, and acellular dermal matrix use, the odds of capsular contracture remained significantly elevated for submuscular DTI (OR 9.03, 95% CI 3.12–26.12; p<0.001) and for expanders (OR 3.25, 95% CI 1.03–10.26; *p* = 0.044) relative to prepectoral reconstruction. Taken together, these data suggest that, within the PMRT setting, prepectoral reconstruction confers a more favourable complication profile, most notably for capsular contracture, than submuscular DTI and, to a lesser extent, expander-based approaches.

These findings support that, among patients receiving PMRT, prepectoral DTI reconstruction does not carry higher major complication rates than expander or submuscular DTI and performs better than submuscular DTI for infection, consistent with the recent trend toward prepectoral DTI even when PMRT is planned.

## Discussion

**Fig. 7:**
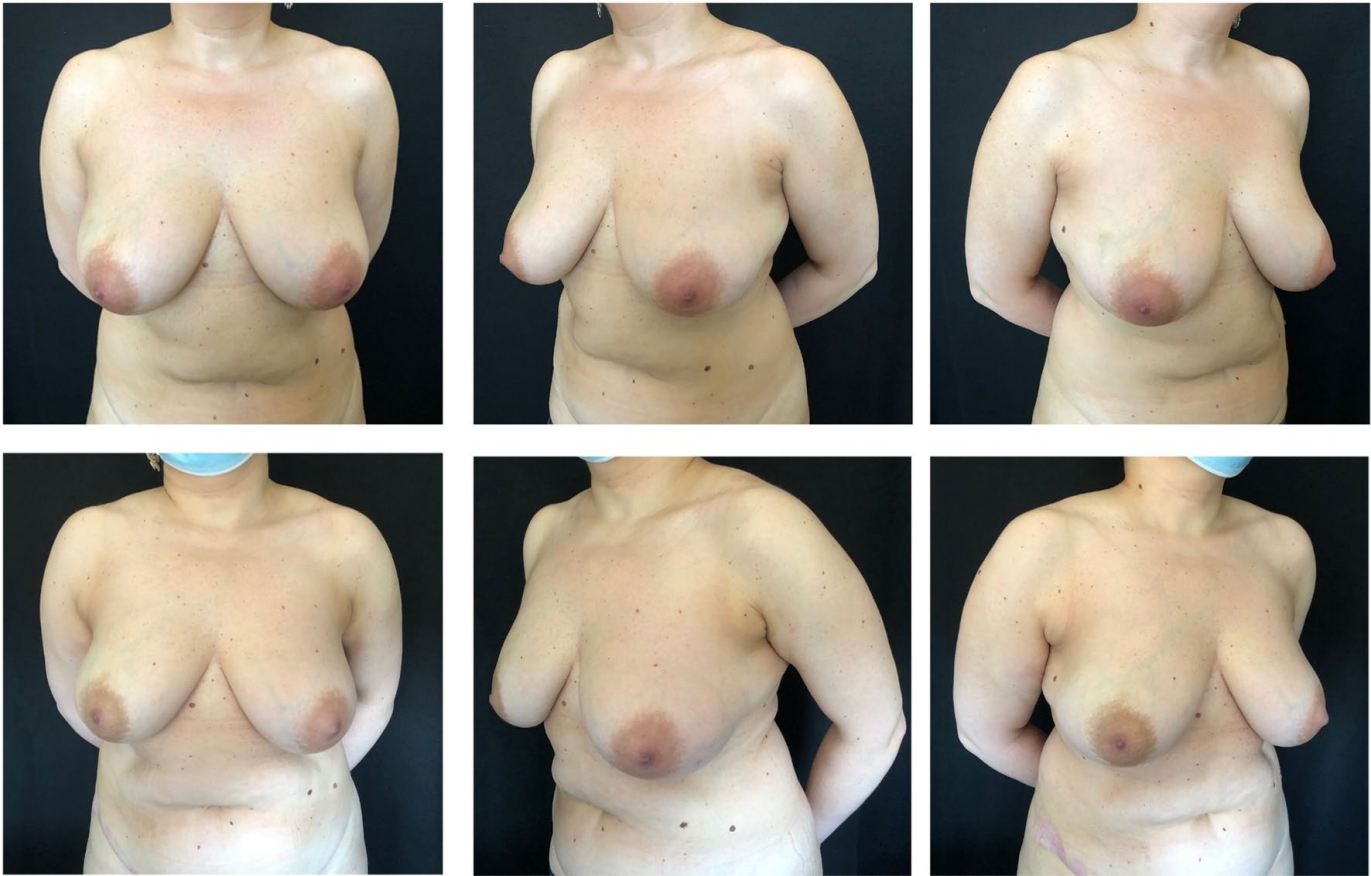
Left nipple-sparing mastectomy performed through inframammary fold incision. Immediate prepectoral direct-to-implant reconstruction using 280-ml polyurethane-coated anatomical implants. Preoperative (upper row) and one-year postoperative outcome (lower row).

**Fig. 8:**
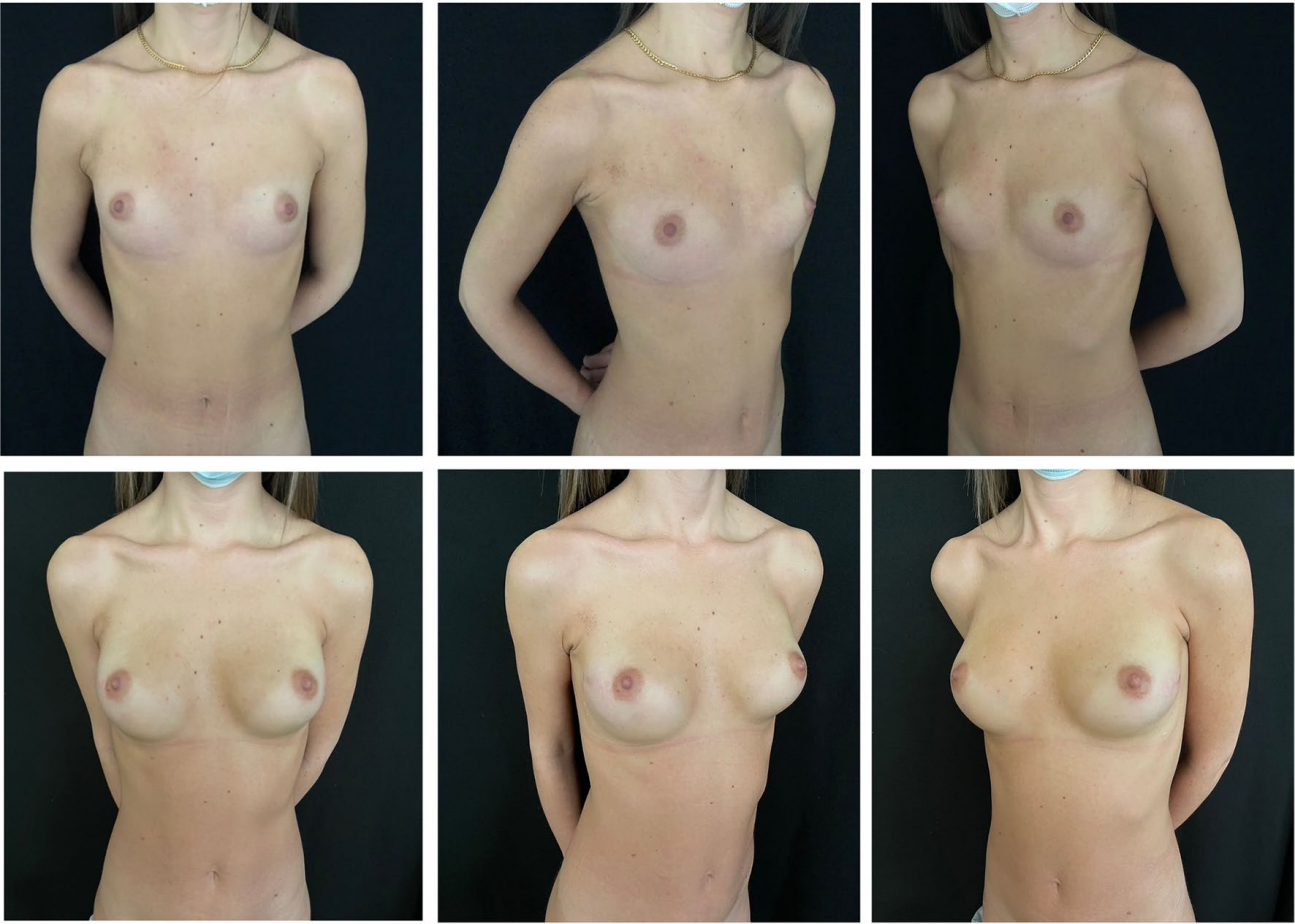
Bilateral nipple-sparing mastectomy performed through lateral radial incisions followed by immediate submuscular direct-to-implant reconstruction with 240-ml micro-textured anatomical implants. Preoperative (upper row) and one-year postoperative result (lower row).

This study proposes a clinically grounded, data-driven pathway for implant-based breast reconstruction in the setting of nipple-sparing and skin-sparing mastectomy. The first gate is flap vascularity. When mastectomy flaps are well perfused, outcomes in our cohort support placing the implant in the prepectoral plane (Fig. 7); when flaps are poorly vascularised, submuscular direct-to-implant reconstruction is, in most cases, the preferred solution (Fig. 8). Clinically, vascularity is often reflected by flap thickness [52]. In our data, thickness was approximately 1.0 cm when prepectoral reconstruction was possible—therefore in well-vascularised flaps—and about 0.4 cm when vascularisation was inadequate. Technique selection should therefore begin with an assessment of perfusion per se (not merely thickness as a “raw” metric), acknowledging that in practice the vascular assessment is mirrored by the measured thickness of the mastectomy flaps.

The second gate is the oncologic plan for postmastectomy radiotherapy, or a prior course of radiotherapy to the involved chest/hemithorax.

**Fig. 9:**
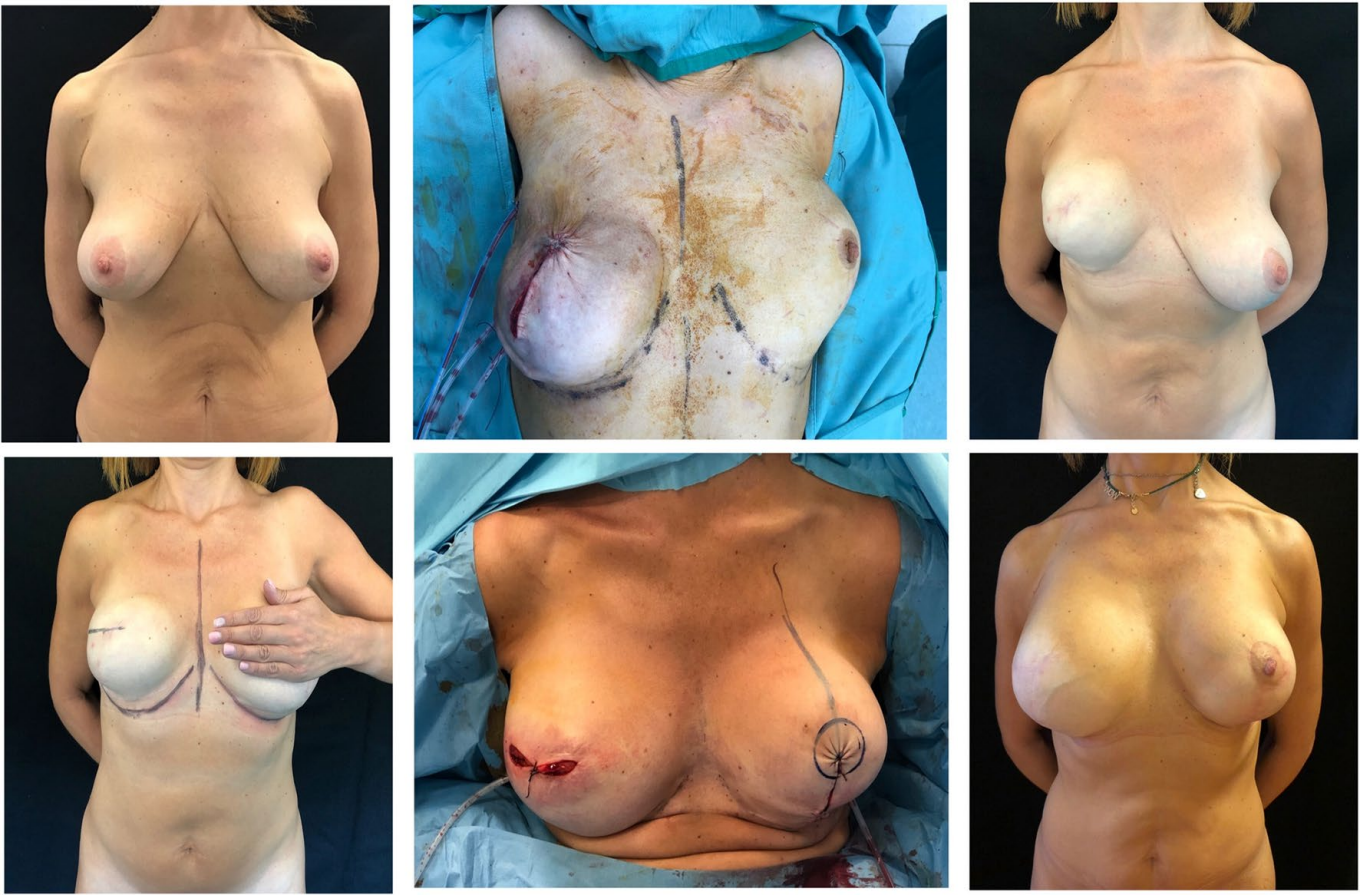
Two-stage implant-based breast reconstruction. First row: Right skin-sparing mastectomy and placement of a tissue expander filled with 230 ml of saline. Second row: Expander exchange to a 450-ml anatomical polyurethane-coated implant and contralateral augmentation with a 265-ml anatomical polyurethane-coated implant and 110g breast reduction.

If radiotherapy is planned, autologous procedures remain the option associated with the most favourable outcomes in this scenario, although the optimal timing of such reconstruction in patients who are candidates for postoperative radiotherapy is still debated in the literature. [53–56] In the subgroup that actually received PMRT or previous radiotherapy, outcomes with prepectoral reconstruction were not worse than with the expander pathway or submuscular direct-to-implant: the composite of major complications (infection, dehiscence, seroma, haematoma, or implant extrusion) was 13.5% after prepectoral placement versus 8.7% with expanders (*p* = 0.73) and 17.1% with submuscular direct-to-implant (*p* = 0.45); infection was lower after prepectoral than after submuscular direct-to-implant (2.7% vs 14.3%, *p* = 0.020). These results are consistent with the findings reported in literature [57, 58]. Capsular contracture within this radiotherapy subgroup was markedly less frequent with prepectoral reconstruction than with submuscular direct-to-implant on crude analysis and remained lower than both alternatives after multiple-imputation–adjusted logistic regression (odds ratios ≈9.0 for submuscular direct-to-implant and ≈3.3 for expanders, each versus prepectoral). These findings are comparable to previous studies, and we have also demonstrated that lipofilling may have a protective effect against capsular contracture in patients undergoing radiotherapy with prepectoral reconstruction, consistent with other reports highlighting the benefits of fat grafting in reducing fibrotic reactions [59–62]. Previous studies have indicated that fat grafting can reduce fibrosis and improve tissue quality in irradiated areas [63–67]. Taken together, these findings led us to adopt the following rule: if flap quality is adequate, prepectoral reconstruction is acceptable; if flaps are thin or compromised, or multiple risk factors are present, a two-stage expander is preferred (Fig. 9). This choice is reflected in our data by a recent calendar-year trend toward prepectoral reconstruction over two-stage submuscular reconstruction in this patient category.

When radiotherapy is not anticipated, the balance of evidence favours prepectoral reconstruction as the baseline strategy alongside submuscular direct-to-implant, recognised as an excellent option for the patient [17]. Using inverse-probability weighting to address case-mix, submuscular direct-to-implant carried a higher weighted risk of the major-complication composite than prepectoral reconstruction (17.1% vs 7.7%; risk difference +9.4 percentage points, p < 0.001). Patient phenotype then refines the choice: younger age and lower body-mass index independently favoured submuscular direct-to-implant over prepectoral reconstruction (relative risk ratios ≈ 0.96 per year and ≈ 0.72 per kg/m^2^), a finding that does not add conceptually beyond the primary rule because lower body-mass index is often associated with thinner flaps, which, as shown above, reflect poorer vascularisation [52, 68]. Smoking has an important impact on wound healing and local vascularity and therefore behaves as a cautionary modifier in the selection model, pushing decisions toward submuscular reconstruction [17, 68]. Polyurethane surfaces predominated in the prepectoral cohort, micro-textured devices in submuscular groups, and acellular dermal matrix was used sparingly; a signal of higher infection in prepectoral cases receiving acellular dermal matrix is compatible with confounding by indication rather than a causal effect.

**Fig. 10:**
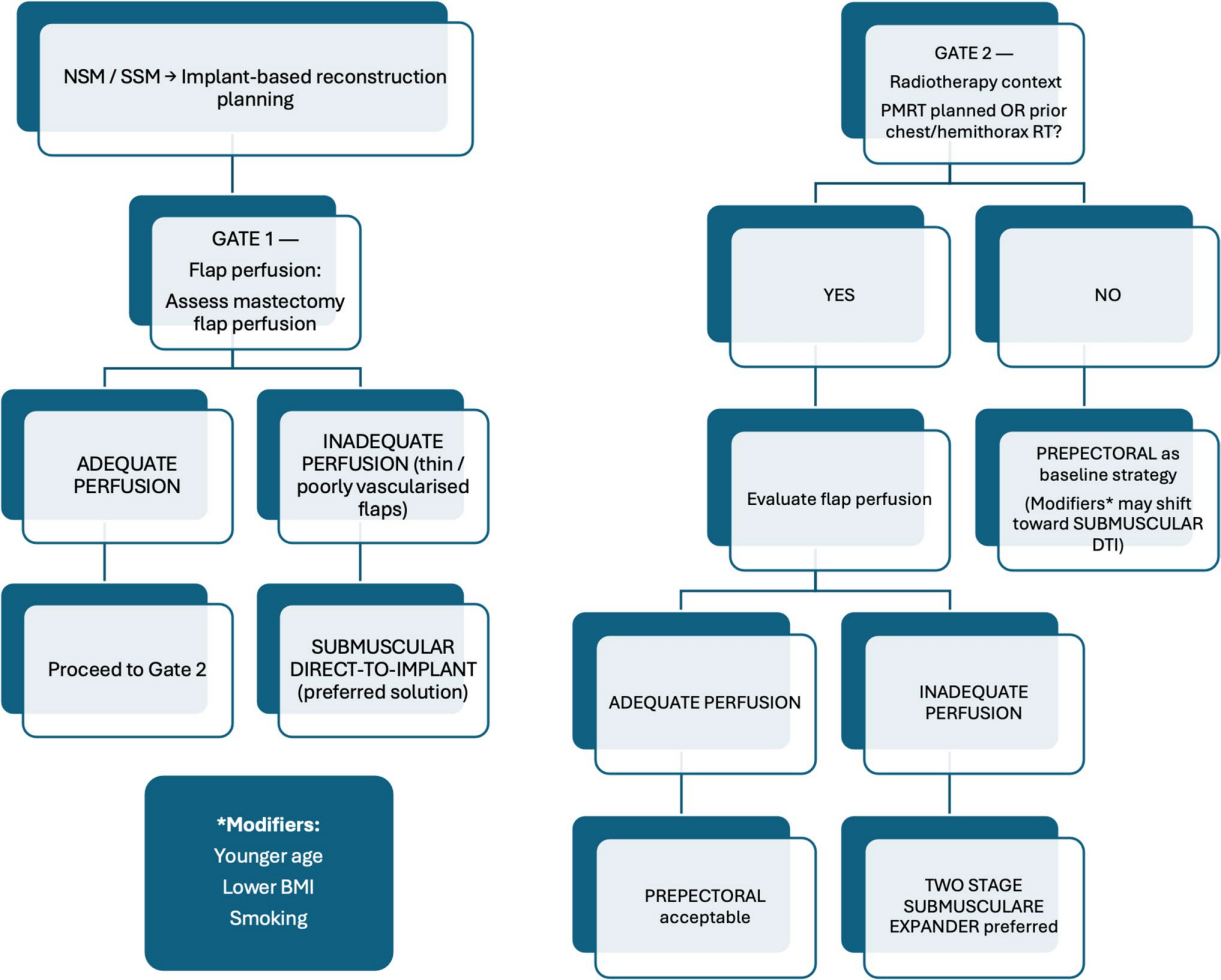
Proposed decision-making algorithm for immediate implant-based breast reconstruction following nipple-sparing (NSM) or skin-sparing mastectomy (SSM).

Practice is evolving in a direction consistent with this algorithm: calendar-year modelling showed a steady increase in prepectoral reconstruction adoption of roughly 30% per year, indicating growing confidence as selection criteria and peri-operative pathways matured. Limitations of this study include the retrospective, single-centre design and the lack of long-term and patient-reported outcomes; nonetheless, the internal coherence between selection signals and risk-adjusted outcomes strengthens inference. In aggregate, these findings support a disciplined, stepwise approach: assess flap vascularisation first; if tissue is robust, prepectoral placement is appropriate; if tissue is thin or poorly perfused, submuscular direct-to-implant reconstruction is preferred; radiotherapy, planned or already delivered, plays a key role in shaping the choice of technique, and patient phenotype together with smoking and postoperative chemotherapy should be treated as risk modifiers (Fig. 10)

## Conclusions

Our findings support a decision-making algorithm for immediate implant-based breast reconstruction grounded in clinical and quantitative evidence. The first step is assessing mastectomy flap vascularity: well-perfused flaps favour prepectoral direct-to-implant placement, whereas thin or poorly vascularized flaps require submuscular direct-to-implant reconstruction. Radiotherapy, whether planned, is a key determinant in selecting the reconstructive approach. In this setting, the choice between prepectoral and two-stage expander should be driven primarily by flap quality, defaulting to an expander when tissue is suboptimal. In addition, patient phenotype, smoking status, and postoperative chemotherapy should be regarded as important risk modifiers. This structured approach, validated by our data, guides surgeons toward safe, individualized selection of reconstruction technique, optimizing both functional and aesthetic outcomes.

## Data Availability

Research data are stored in an institutional repository and will be shared upon request to the corresponding author.

## References

[1] World Cancer Research Fund. Worldwide cancer data. 2022.

[2] Schmauss D, Machens HG, Harder Y. Breast reconstruction after mastectomy. Front Surg. 2015;2:71. 26835456 10.3389/fsurg.2015.00071 PMC4717291

[3] Early Breast Cancer Trialists’ Collaborative Group, Darby S, McGale P, et al. Effect of radiotherapy after breast-conserving surgery on 10-year recurrence and 15-year breast cancer death: meta-analysis of individual patient data for 10,801 women in 17 randomised trials. Lancet. 2011;378(9804):1707–16. 22019144 10.1016/S0140-6736(11)61629-2 PMC3254252

[4] Al-Ghazal SK, Fallowfield L, Blamey RW. Comparison of psychological aspects and patient satisfaction following breast-conserving surgery, simple mastectomy and breast reconstruction. Eur J Cancer. 2000;36(15):1938–43. 11000574 10.1016/s0959-8049(00)00197-0

[5] Panchal H, Matros E. Current trends in postmastectomy breast reconstruction. Plast Reconstr Surg. 2017;140(5S):7S-13S. 29064917 10.1097/PRS.0000000000003941 PMC5722225

[6] Nahabedian MY. Implant-based breast reconstruction following conservative mastectomy: one-stage versus two-stage approach. Gland Surg. 2016;5(1):47–54. 26855908 10.3978/j.issn.2227-684X.2015.06.08 PMC4716858

[7] Simion L, Petrescu I, Chitoran E, Rotaru V, Cirimbei C, Ionescu SO, et al. Breast reconstruction following mastectomy for breast cancer or prophylactic mastectomy: therapeutic options and results. Life (Basel). 2024;14(1):138. 38255753 10.3390/life14010138 PMC10821438

[8] Murphy BD, Kerrebijn I, Farhadi J, Masia J, Hofer SOP. Indications and controversies for abdominally-based complete autologous tissue breast reconstruction. Clin Plast Surg. 2018;45(1):83–91. 29080663 10.1016/j.cps.2017.08.007

[9] Opsomer D, van Landuyt K. Indications and controversies for nonabdominally-based complete autologous tissue breast reconstruction. Clin Plast Surg. 2018;45(1):93–100. 29080664 10.1016/j.cps.2017.08.012

[10] Al Qurashi AA, et al. Efficacy of exclusive fat grafting for breast reconstruction: an updated systematic review and meta-analysis. Aesthetic Plast Surg. 2024;48(23):4979–85. 38772941 10.1007/s00266-024-03978-3

[11] Fabiocchi L, Lucattelli E, Cattin F, et al. Reverse expansion for breast reconstruction after skin-sparing and nipple-sparing mastectomy: our first 100 cases. Plast Reconstr Surg. 2023;11(4):e4915. 10.1097/GOX.0000000000004915 PMC10069859 37020986

[12] Chang DW, Barnea Y, Robb GL. Effects of an autologous flap combined with an implant for breast reconstruction: an evaluation of 1000 consecutive reconstructions of previously irradiated breasts. Plast Reconstr Surg. 2008;122(2):356–62. 18626350 10.1097/PRS.0b013e31817d6303

[13] Gougoutas AJ, Anderson BO. One-stage versus two-stage breast reconstruction: prudence in surgical decision-making. Lancet Oncol. 2017;18(2):166–7. 28012976 10.1016/S1470-2045(16)30673-8

[14] Ter Louw RP, Nahabedian MY. Prepectoral breast reconstruction. Plast Reconstr Surg. 2017;140(5S):51S-59S. 29064922 10.1097/PRS.0000000000003942

[15] Ribuffo D, Berna G, De Vita R, et al. Dual-plane retropectoral versus prepectoral DTI breast reconstruction: an Italian multicenter experience. Aesthet Plast Surg. 2020;45:51–60. 10.1007/s00266-020-01892-y PMC7886728 32860077

[16] American Society of Plastic Surgeons. Plastic surgery statistics report 2023. 2023.

[17] Masia J, et al. Prepectoral reconstruction with ADM: iBAG multicenter study. Eur J Surg Oncol. 2021;47(1):123–31.32561204

[18] Cattelani L, Polotto S, Arcuri MF, et al. One-step prepectoral breast reconstruction with dermal matrix-covered implant compared to submuscular implantation: functional and cost evaluation. Clin Breast Cancer. 2018;18(4):e703–11. 29275104 10.1016/j.clbc.2017.11.015

[19] Maruccia M, Di Taranto G, Onesti MG. One‐stage muscle‐sparing breast reconstruction in elderly patients: a new tool for retaining excellent quality of life. Breast J. 2018;24(2):180–3. 28703387 10.1111/tbj.12860

[20] Reitsamer R, Peintinger F, Klaassen-Federspiel F, Sir A. Prepectoral direct-to-implant breast reconstruction with complete ADM or synthetic mesh coverage-36-months follow-up in 200 reconstructed breasts. Breast. 2019;48:32–7. 31491673 10.1016/j.breast.2019.08.002

[21] Nolan IT, Farajzadeh MM, Boyd CJ, Bekisz JM, Gibson EG, Salibian AA. Do we need acellular dermal matrix in prepectoral breast reconstruction? A systematic review and meta-analysis. J Plast Reconstr Aesthet Surg. 2023;86:251–60. 37793198 10.1016/j.bjps.2023.09.042

[22] Berna G, Cawthorn SJ, Papaccio G, Balestrieri N. Evaluation of a novel breast reconstruction technique using the Braxon® acellular dermal matrix: a new muscle‐sparing breast reconstruction. ANZ J Surg. 2017;87(6):493–8. 25266930 10.1111/ans.12849

[23] Manrique OJ, et al. Prepectoral two-stage implant-based breast reconstruction with and without acellular dermal matrix: do we see a difference? Plast Reconstr Surg. 2020;145:263e–72e. 31985613 10.1097/PRS.0000000000006442

[24] Franceschini G, Masetti R. Immediate implant-based breast reconstruction with acellular dermal matrix after conservative mastectomy: Can a more effective alternative be used in the near future? Eur J Surg Oncol. 2020;47:1225–6. 33023794 10.1016/j.ejso.2020.09.037

[25] De Vita R, Buccheri EM, Villanucci A, Pozzi M. Breast reconstruction actualized in nipple-sparing mastectomy and direct-to-implant, prepectoral polyurethane positioning: early experience and preliminary results. Clin Breast Cancer. 2019;19:e358–63. 30691930 10.1016/j.clbc.2018.12.015

[26] Franceschini G, et al. Immediate prosthetic breast reconstruction after nipple-sparing mastectomy: traditional subpectoral technique versus direct-to-implant prepectoral reconstruction without acellular dermal matrix. J Pers Med. 2021;11(2):153. 33671712 10.3390/jpm11020153 PMC7926428

[27] Blondeel PN, et al. Shaping the breast in aesthetic and reconstructive breast surgery: an easy three-step principle. Plast Reconstr Surg. 2009;123:455–62. 19182601 10.1097/PRS.0b013e3181954cc1

[28] Hunsicker LM, Ashikari AY, Berry C, Koch RM, Salzberg CA. Short-term complications associated with acellular dermal matrix–assisted direct-to-implant breast reconstruction. Ann Plast Surg. 2017;78(1):35–40. 10.1097/SAP.0000000000000742. 26849284 10.1097/SAP.0000000000000742

[29] Lawrence WT, Talbot TL, Norton JA. Preoperative or postoperative doxorubicin hydrochloride (adriamycin): which is better for wound healing? Surgery. 1986;100(1):9–13. 3726767

[30] Devereux DF, Thibault L, Boretos J, Brennan MF. The quantitative and qualitative impairment of wound healing by adriamycin. Cancer. 1979;43(3):932–8. 427732 10.1002/1097-0142(197903)43:3<932::aid-cncr2820430322>3.0.co;2-a

[31] Hu YY, Weeks CM, In H, Dodgion CM, Golshan M, Chun YS, et al. Impact of neoadjuvant chemotherapy on breast reconstruction. Cancer. 2011;117(13):2833–41. 21264833 10.1002/cncr.25872 PMC3164976

[32] Mitchem J, Herrmann D, Margenthaler JA, Aft RL. Impact of neoadjuvant chemotherapy on rate of tissue expander/implant loss and progression to successful breast reconstruction following mastectomy. Am J Surg. 2008;196(4):519–22. 18809054 10.1016/j.amjsurg.2008.06.016

[33] Sabitovic A, Trøstrup H, Damsgaard TE. The impact of neoadjuvant chemotherapy on surgical outcomes following autologous and implant-based immediate breast reconstruction: a systematic review and meta-analysis. J Plast Reconstr Aesthet Surg. 2023;87:17–23. 10.1016/j.bjps.2023.09.048. 37804643 10.1016/j.bjps.2023.09.048

[34] Srinivasa DR, Garvey PB, Qi J, Hamill JB, Kim HM, Pusic AL, et al. Direct-to-implant versus two-stage tissue expander/implant reconstruction: 2-year risks and patient-reported outcomes from a prospective, multicenter study. Plastic Reconstruct Surg. 2017;140(5):869–77. 10.1097/PRS.0000000000003748. 10.1097/PRS.0000000000003748 PMC5902733 29068918

[35] Qu FL, Wu SY, Li JJ, Shao ZM. Ipsilateral breast tumor recurrence after breast-conserving surgery: insights into biology and treatment. Breast Cancer Res Treat. 2023;202(2):215–20. 37528263 10.1007/s10549-023-07071-2

[36] Qu FL, Mao R, Liu ZB, Lin CJ, Cao AY, Wu J, et al. Spatiotemporal patterns of loco-regional recurrence after breast-conserving surgery. Front Oncol. 2021;30:11:690658. 10.3389/fonc.2021.690658 PMC8435899 34527574

[37] Singh M, Alavi A, Wong R, Akita S. Radiodermatitis: a review of our current understanding. Am J Clin Dermatol. 2016;17(3):277–92. 27021652 10.1007/s40257-016-0186-4

[38] Sinik LM, Collins MS. Challenges in autologous breast reconstruction: a review of recommendations. J Clin Med. 2024;13(4):971. 38398283 10.3390/jcm13040971 PMC10889312

[39] Liew B, Southall C, Kanapathy M, Nikkhah D. Does post-mastectomy radiation therapy worsen outcomes in immediate autologous breast flap reconstruction? A systematic review and meta-analysis. J Plast Reconstr Aesthet Surg. 2021;74(12):3260–80. 34565703 10.1016/j.bjps.2021.08.005

[40] Aliu O, Zhong L, Chetta MD, et al. Comparing health care resource use between implant and autologous reconstruction of the irradiated breast: a national claims-based assessment. Plast Reconstr Surg. 2017;139(6):1224e–31e. 10.1097/PRS.0000000000003336. 28538545 10.1097/PRS.0000000000003336 PMC5448285

[41] Coudé Adam H, Frisell A, Liu Y, et al. Effect of radiotherapy on expanders and permanent implants in immediate breast reconstruction: long-term surgical and patient-reported outcomes in a large multicentre cohort. Br J Surg. 2021;108(12):1474–82. 10.1093/bjs/znab333. 34694356 10.1093/bjs/znab333

[42] van Huizum MA, Hage JJ, Russell NS, Rutgers E, Woerdeman LAE. Combined skin-sparing mastectomy and immediate implant-based breast reconstruction: outcome following mantle field irradiation versus outcome following whole-breast irradiation. J Plast Reconstr Aesthet Surg. 2022;75(1):94–103. 10.1016/j.bjps.2021.08.003. 34483080 10.1016/j.bjps.2021.08.003

[43] de Boniface J, Coudé Adam H, Frisell A, et al. Long-term outcomes of implant-based immediate breast reconstruction with and without radiotherapy: a population-based study. Br J Surg. 2022;109(11):1107–15. 10.1093/bjs/znac283. 35949111 10.1093/bjs/znac283 PMC10364787

[44] Sekiguchi K, Kawamori J, Yamauchi H. Breast reconstruction and postmastectomy radiotherapy: complications by type and timing and other problems in radiation oncology. Breast Cancer. 2017;24(4):511–20. 10.1007/s12282-017-0754-3. 28108966 10.1007/s12282-017-0754-3

[45] Zeng L, Xie XQ, Luo T, et al. Progress of radiotherapy after breast-conserving surgery combined with silicone prosthesis reconstruction. Eur Rev Med Pharmacol Sci. 2021;25(5):2193–8. 10.26355/eurrev_202103_25210. 33755956 10.26355/eurrev_202103_25210

[46] Zhang X, Ning S, Zhang Y. Complications after prepectoral versus subpectoral breast reconstruction in patients receiving postmastectomy radiation therapy: a systematic review and meta-analysis. Aesthetic Plast Surg. 2024;48(21):4421–9. 10.1007/s00266-024-04096-w. 38700543 10.1007/s00266-024-04096-w

[47] Ho AL, Bovill ES, Macadam SA, et al. Postmastectomy radiation therapy after immediate two-stage tissue expander/implant breast reconstruction: a University of British Columbia perspective. Plast Reconstr Surg. 2014;134(1):1e–10e. 10.1097/PRS.0000000000000292. 25028850 10.1097/PRS.0000000000000292

[48] Carlson GW. Should we be doing implant-based breast reconstruction in the setting of radiotherapy? Ann Surg Oncol. 2014;21(7):2122–3. 10.1245/s10434-014-3491-2. 24743903 10.1245/s10434-014-3491-2

[49] Yun JH, Diaz R, Orman AG. Breast reconstruction and radiation therapy. Cancer Control. 2018;25(1):1073274818795489. 10.1177/1073274818795489. 30132338 10.1177/1073274818795489 PMC6108018

[50] Harless CA, Jacobson SR. Tailoring through technology: a retrospective review of a single surgeon’s experience with implant-based breast reconstruction before and after implementation of laser-assisted indocyanine green angiography. Breast J. 2016;22(3):274–81. 10.1111/tbj.12576. 26899399 10.1111/tbj.12576

[51] Newman MI, Samson MC, Tamburrino JF, Swartz KA. Intraoperative laser-assisted indocyanine green angiography for the evaluation of mastectomy flaps in immediate breast reconstruction. J Reconstr Microsurg. 2010;26(7):487–92. 10.1055/s-0030-1261701. 20539977 10.1055/s-0030-1261701

[52] Pagliara D, Montella RA, Garganese G, Bove S, Costantini M, Rinaldi PM, et al. Improving decision-making in prepectoral direct-to-implant reconstruction after nipple sparing mastectomy: the key role of flap thickness ratio. Clin Breast Cancer. 2023;23(2):e37–44. 10.1016/j.clbc.2022.11.007. 36610826 10.1016/j.clbc.2022.11.007

[53] Thiruchelvam PTR, Leff DR, Godden AR, Cleator S, Wood SH, Kirby AM, et al. Primary radiotherapy and deep inferior epigastric perforator flap reconstruction for patients with breast cancer (PRADA): a multicentre, prospective, non-randomised, feasibility study. Lancet Oncol. 2022;23(5):682–90. 10.1016/S1470-2045(22)00145-0. 35397804 10.1016/S1470-2045(22)00145-0 PMC9630150

[54] Ren Y, Yu Y, Xu K, Li Z, Wang X. Meta-analysis of immediate implant-based breast reconstruction versus autologous breast reconstruction in the setting of PMRT. Aesthet Plast Surg. 2024;48(10):1940–8. 10.1007/s00266-023-03430-y. 10.1007/s00266-023-03430-y 37380747

[55] Heiman AJ, Gabbireddy SR, Kotamarti VS, Ricci JA. A meta-analysis of autologous microsurgical breast reconstruction and timing of adjuvant radiation therapy. J Reconstr Microsurg. 2021;37(4):336–45. 10.1055/s-0040-1716846. 32957153 10.1055/s-0040-1716846

[56] Kalmar CL, Montorfano L, Thayer WP, Kassis S, Higdon KK, Perdikis G. Timing of autologous tissue breast reconstruction does not affect free flap failure. Ann Plast Surg. 2024;92(6):663–6. 10.1097/SAP.0000000000003900. 38717156 10.1097/SAP.0000000000003900

[57] Zhang X, Ning S, Zhang Y. Complications after prepectoral versus subpectoral breast reconstruction in patients receiving postmastectomy radiation therapy: a systematic review and meta-analysis. Aesthet Plast Surg. 2024;48(21):4421–9. 10.1007/s00266-024-04096-w. 10.1007/s00266-024-04096-w 38700543

[58] Salgarello M, Pino VS, Taraschi F, Visconti G, Cellini F, Marazzi F, et al. The impact of postmastectomy radiation therapy on immediate prepectoral reconstruction with polyurethane-coated implants. Aesthetic Plast Surg. 2025. 10.1007/s00266-025-05119-w. 40841797 10.1007/s00266-025-05119-w PMC12916999

[59] Sobti N, Weitzman RE, Nealon KP, Jimenez RB, Gfrerer L, Mattos D, et al. Evaluation of capsular contracture following immediate prepectoral versus subpectoral direct-to-implant breast reconstruction. Sci Rep. 2020;10(1):1137. 10.1038/s41598-020-58094-4. 31980737 10.1038/s41598-020-58094-4 PMC6981172

[60] Sinnott CJ, Persing SM, Pronovost M, Hodyl C, McConnell D, Ott Young A. Impact of postmastectomy radiation therapy in prepectoral versus subpectoral implant-based breast reconstruction. Ann Surg Oncol. 2018;25(10):2899–908. 10.1245/s10434-018-6602-7. 29978367 10.1245/s10434-018-6602-7

[61] Barone Adesi L, Taraschi F, Macrì G, Scardina L, Di Leone A, Franceschini G, et al. Fat grafting and prepectoral prosthetic reconstruction with polyurethane-covered implants: protective role against adjuvant radiotherapy. J Clin Med. 2024;13(17):4982. 10.3390/jcm13174982. 39274192 10.3390/jcm13174982 PMC11396578

[62] Pagliara D, Vitagliano S, Mangialardi ML, Pino V, Santoro A, Mulè A, et al. The role of fat grafting on contracted breast implant capsules: a retrospective comparative histological and immunohistochemical study. J Plast Reconstr Aesthet Surg. 2021;74:2975–85. 10.1016/j.bjps.2021.09.035 34838502

[63] Borrelli MR, Patel RA, Adem S, Diaz Deleon NM, Shen AH, Sokol J, et al. Grafted fat enriched with CD74+ adipose-derived stromal cells reduces radiation-induced fibrosis. Stem Cells Transl Med. 2020;9:1401–8. 32563212 10.1002/sctm.19-0317 PMC7581454

[64] Borrelli MR, Diaz Deleon NM, Adem S, Patel RA, Mascharak S, Shen AH, et al. Fat grafting rescues radiation-induced joint contracture. Stem Cells. 2020;38:382–9. 31793745 10.1002/stem.3115 PMC8033730

[65] Gerth DJ, King TW, Rabach LA. Fat chance: the rejuvenation of irradiated skin. Plast Reconstr Surg Glob Open. 2019;7:e2120. 30881833 10.1097/GOX.0000000000002092 PMC6416118

[66] Huang S, Kates M, Perlman S. Increasing fat graft retention in irradiated tissue after preconditioning with external volume expansion. Plast Reconstr Surg. 2024;134:75–85. 10.1097/PRS.0000000000006372 31577660

[67] Chinnapaka S, Yang KS, Surucu Y, Bengur FB, Arellano JA, Tirmizi Z, et al. Human adipose ECM alleviates radiation-induced skin fibrosis via adipose progenitor cell recruitment and extracellular matrix remodeling. iScience. 2023;26:106756. 37705953 10.1016/j.isci.2023.107660 PMC10495661

[68] Frey JD, Salibian AA, Choi M, Karp NS. Mastectomy flap thickness and complications in nipple-sparing mastectomy: objective evaluation using magnetic resonance imaging. Plast Reconstr Surg Glob Open. 2017;5(8):e1439. 10.1097/GOX.0000000000001439. 28894660 10.1097/GOX.0000000000001439 PMC5585433

